# Supplementary material for: Differences between Scaly-sided Merganser (Mergus squamatus) and Common Merganser (M. merganser) feather microstructure
Source: PLoS One. 2026 Jan 27;21(1):e0319144. doi: 10.1371/journal.pone.0319144 (PMC12843524; doi:10.1371/journal.pone.0319144)
Supplement: Attachment — Submitted filename: Response to Reviewers.docx [file pone.0319144.s003.docx]

**Response to Reviewers**

**Reviewer #1：The authors stated that the microstructures present in the breast feathers, flank feathers, rectrices, and down feathers are similar. However, they also noted that "Notably, variations were observed in the length of the base handle from distal barbules in breast feathers." It is surprising to see how species identification could be scientifically determined solely based on the length and distance of feather microstructures, especially when all selected feather types exhibit similar microstructures. Given that measurements will vary according to age and feather location, the authors' claim may be invalid. Without the presence of a distinct microstructure, species identification between the two studied species would not be feasible.**

We thank the reviewer for their comments and for raising an important concern regarding the species identification based on microstructural differences in feathers. We fully acknowledge that relying merely on the length and distance variations of feather microstructural features, without considering the overall structural characteristics, may not always yield reliable results for species identification—especially when different feather types exhibit similar microstructural patterns.

In this study, we randomly collected feather samples of the Scaly-sided Merganser after observing a group of them molting and leaving. As a result, we were unable to classify the Scaly-sided Merganser samples by age and instead analyzed all the samples together. Specifically, 20 feathers of different types were randomly selected, including flank contour feathers, rectrices, and down feathers. In addition, we further expanded the sample size of Common Merganser feathers. These feathers were procured from diverse sources: an adult female Common Merganser specimen preserved at Jilin Normal University in Siping, Jilin Province, China; an adult male Common Merganser carcass donated by a volunteer from Shenyang, Liaoning Province, China; and a large quantity of feathers supplied by volunteers in Fuzhou, Fujian Province, China, with information on the age and exact number of individuals unknown for Common Merganser feather samples. Finally, three feathers per type were collected from each individual and randomly selected 14 feathers of each type from the Common Merganser samples provided by Fuzhou volunteer. These samples constituted the basis for analysis.

However, we still found some microstructural characteristics that differed significantly between the two species. Based on these findings, we speculate that these differences were likely to stem from species-specific traits rather than age-related factors. Furthermore, stepwise discriminant analysis further validated the discriminatory power of all microstructural characteristics. For contour feathers, incorporating the base length into the model achieved a 56.9% correct classification rate. In rectrices, the hooklet number and prong length emerged as key discriminators, with a correct classification rate of 91.3%. Most notably, the barbule length, node number, and inter-node distance of down feathers demonstrated exceptional discriminative capabilities, attaining a perfect 100% correct classification rate. Consequently, the barbule length, node number, and inter-node distance of down feathers, may be considered as crucial morphological markers for differentiating the Scaly-sided Merganser from the Common Merganser.

Species identification using the microstructural characteristics of feathers offers several advantages, including accelerating laboratory species identification, reducing costs, and enhancing work efficiency. To further advance research in this field, achieving accurate identification of feathers of unknown origin is a key goal. Future studies should consider expanding the sample scope to include more individuals of varying ages and sexes, thereby strengthening the generalizability of the research findings. Moreover, integrating multidisciplinary techniques such as genomics and bioinformatics to explore the genetic underpinnings of feather microstructural characteristics will provide a more solid theoretical foundation for species identification.

Thank you again for your valuable input.

**Reviewer #2：Overview: It is clear that the authors have dedicated much time and effort towards outlining and distinguishing these two species’ morphological barbule characters. There do appear to be differences in quite a few of these characters. However, I would heavily caution this interpretation on the basis of only one individual (Common Merganser) compared to what appears to be approx. 20 Scaly-sided Merganser individuals. It’s unclear as to which of the characters the authors recommend could/should be used to accurately discern Scaly-sided from Common Merganser – or whether all of the characters for multiple feather types are required to be taken together to accurately distinguish these two species from one another. This would be helpful for a practical application of these data, as the authors talk about it being useful for. And finally, a suggestion that a methods figure would greatly improve the ability of another scientist to understand these measurements and to be able to replicate these measurements on SEM images as this author.**

We sincerely appreciate your comprehensive review and constructive insights.

First, we fully understand your concern regarding the interpretation based on only one individual (Common Merganser) initially. It is indeed a risky approach. Given that the Common Merganser is also a protected species, acquiring a large number of samples poses significant challenges. To address this issue, we obtained an adult male Common Merganser carcass from a volunteer in Shenyang, Liaoning Province, China. We also received multiple Common Merganser feathers from a volunteer in Fuzhou, Fujian Province, China, with information on the age and exact number of individuals unknown for Common Merganser feather samples. Additionally, the exact number of individual Scaly-sided Mergansers involved in this study cannot be confirmed. Regarding the collection of Scaly-sided Merganser feathers from Longhu Mountain in Jiangxi Province, China, we observed a group of over 20 Scaly-sided Mergansers through binoculars during the moulting period. To minimize any disturbance, we randomly collected feathers only after the birds had left the site. Therefore, we were unable to determine the exact number of individuals, nor could we classify the samples based on age. Given these limitations, we combined all the collected samples for analysis and randomly selected 20 samples of different feather types, including contour feathers, rectrices, and down feathers.

Secondly, several microstructural characteristics of feathers exhibited significant variations between the two species. In rectrices, significant variations were observed in the prong length, base length, hooklet number, and prong number of distal barbules. Similarly, down feathers exhibited marked differences in the node number, inter-node distance, internode width, and barbule length of downy barbules. Stepwise discriminant analysis further validated the discriminatory power of all microstructural characteristics. For contour feathers, incorporating the base length into the model achieved a 56.9% correct classification rate. In rectrices, the hooklet number and prong length emerged as key discriminators, with a correct classification rate of 91.3%. Most notably, the barbule length, node number, and inter-node distance of down feathers demonstrated exceptional discriminative capabilities, attaining a perfect 100% correct classification rate. Consequently, the barbule length, node number, and inter-node distance of down feathers, may be considered as crucial morphological markers for differentiating the Scaly-sided Merganser from the Common Merganser.

Finally, to improve the clarity of our methods, we have added Table 1, which clearly describes all measurement methods and parameters. We have also updated Figs. 1, 2, and 3 with corresponding labels to better illustrate the measurement process, aiming to facilitate other scientists in understanding and replicating our measurements on SEM images.

Thank you again for your thorough review and constructive feedback.

1 **Abstract: Can you add the take home of what individual morphological barbule characters are recommended to be used to distinguish these two species?**

Additions have been made. “all the microstructural features of down feathers, particularly barbule length, may be considered as crucial morphological markers for differentiating the two species.”

2 **Line 45 – Add Laybourne after Roxie to indicate who you are referring to**

**Line 46- A notable example of what? Might add that this is a notable example of the usefulness of these characters/notable example of the practical application of these characters etc.**

**Line 46 – Change ‘common starling’ to ‘European Starling’ (capitalization and common English name change.**

Additions and modifications have been made. “A notable example of the practical application of these characters is Roxie Laybourne's work, where she identified the European Starling (*Sturnus vulgaris*) as the cause of an aviation accident by analyzing feathers found in the wreckage[3].”

3 **Line 53 – concern regarding – maybe replace this English phrasing to: interest in. I don’t think there are concerns regarding these topics, but rather people heavily invested in examining and detailing these aspects of Scaly-sided Merganser biology.**

Modifications have been made. “Consequently, there is substantial interest in various aspects of Scaly-sided Merganser, including population size [10–12], habitat selection [13,14], genetic diversity [15], migration connections [16], and breeding ecology [17].”

4 **Line 56 – ‘to address these issues – similar adjustment as above. Something like ‘answer questions related to these topics.’ Instead, is a better overview of the goal.**

Modifications have been made. “In addition to field observations, molecular biology and isotope tracing are often used to answer questions related to these topics.”

5 **Line 57- direct capture OF blood. I think you’re referring to the direct capture of birds in order to take samples from them in which case, replace the word ‘of’ with ‘in order to take’ and that should more accurately describe what is difficult.**

Modifications have been made. “However, due to the rarity and high vigilance of Scaly-sided Mergansers, direct capture in order to take blood or tissue samples is becoming increasingly challenging.”

6 **Line 60- classified as a (not the) species**

Modifications have been made. “It is noteworthy that Common Merganser (*M. merganser*), classified as a species of least concern according to the IUCN Red List, is distinguished as the largest and most widely distributed species within the genus *Mergus* in China[18].”

7 **Line 64 - …as ‘molted/found/undetermined’ feather samples collected. Typically feather samples don’t come from both species, but feather samples collected from an undetermined locality or found feather samples could come from both species or molted feathers found/taken from the ground are all feather samples that could come from both species.**

Modifications have been made. “This presents a challenge for ecologists, as feather samples, whether molted, found, or from an undetermined locality, may originate from both species.”

8 **Line 71 – morphological differentiation, in theory, wouldn’t be imperative but it does become an incredibly useful tool before sequencing DNA. I’d just change that particular word (imperative).**

Modifications have been made. “Therefore, morphological differentiation of feather samples is an incredibly useful tool before sequencing DNA.”

9 **Materials/Methods:** **What was the condition of the feathers examined? Were more degraded feathers more difficult but still possible or impossible to see barbule characters?**

Since the birds were observed moulting with binoculars and collected immediately when they left, the feathers examined were in good condition and not degraded. This has been written under Materials/Methods.

10 **Line 83 – How were approx. 20 individuals determined for Scaly-sided Mergansers? Was this genetically verified as 20 individuals? By a repeat number of parts (for example there were at least 20 right outermost primaries found/sampled? Sample size for each of the feather types per species.**

A group of more than 20 Scaly-sided Mergansers was observed through binoculars during the moulting period. However, to minimize disturbance, feathers were only collected after the birds had left the site. As a result, the exact number of individual Scaly-sided Mergansers involved in this study cannot be confirmed. From the collected feathers, 20 feathers of different types were randomly selected, including flank contour feathers, rectrices, and down feathers.

Additionally, feathers of Common Merganser were obtained from the following sources: an adult female Common Merganser specimen from the collection of Jilin Normal University in Siping City, Jilin Province, China; an adult male Common Merganser carcass provided by a volunteer from Shenyang City, Liaoning Province, China; and multiple feathers of the Common Merganser provided by a volunteer from Fuzhou City, Fujian Province, China. Finally, three feathers of each type were collected from each individual. Moreover, 14 feathers of each type were randomly chosen from the Common Merganser feather samples provided by volunteers.

Hence, the sample size was 20 per species per feather type.

11 **Line 84 – Referred as ‘classified according to Featherbase’. What was being classified and how was Featherbase used to classify them as such. I think just a general clarification of what they were classified as, using Featherbase will help readers understood how it was used. Am I understanding this correctly that there was only ONE Common Merganser sampled and approx. 20 Scaly-sided Mergansers sampled?**

Yes, you are correct. We sampled one Common Merganser and approximately 20 Scaly - sided Mergansers.

There is a feather specimen of an adult Scaly-sided Merganser in Featherbase. We performed a simple classification of the randomly collected feathers based on Featherbase. However, after further consideration, we revised the paper, as distinguishing the contour feathers may not be accurately done solely based on Featherbase. Therefore, we removed the analysis of the breast feathers. Since the Scaly-sided Merganser is known for its fish-scale-like flank feathers, which can be easily identified from feather samples, we retained the observation and analysis of the contour feathers of the flank.

12 **Line 88 – Why was the middle part of the feather vane sampled when most barbule characters are more well defined at the base of the feather? Were pennaceous barbs used? The plumulaceous feathers at the base of the feather vane? Distal, intermediate, basal or umbilical plumulaceous regions of the feather are typically used to describe which feather barbs were examined.**

The terminology used to describe the feather sections was inaccurate and has been revised. Additionally, adjustments have been made to the Materials and Methods section.

13 **Line 172 – when ‘slender morphology’ is used – what is being meant? I think you might want to use more descriptors to more accurately describe the morphology.**

The down feathers section has been revised and adjusted.

14 **Table 1 – The measurements taken, is there any inter-observer differences in measurements? How were measurements taken? Was there a program used to do the measuring? These measurements aren’t super intuitive, and the terminology used differs slightly from Chandler and Carla Dove’s previous and clearly defined characters. For example, the “Length of base nodular” – is this referring to the length of the most basal node or is it referring to the length of the basal cell (before the basal node). So, to make it simple I think making an example figure of a feather / barbule etc and showing the different measurements taken would be incredibly useful to visualize each and allow for someone else to repeat the study/take the same measurements in the same fashion.**

Indeed, the terminology used was somewhat inaccurate, and these measurements aren't very intuitive. We have revised the terminology throughout the paper. In Table 1, all the measurement methods and parameters are clearly explained, and corresponding labels are provided in Figs 1, 2 and 3 to better illustrate the measurement process.

15 **Line 210 – common mergers should be “Common Mergansers”**

Modifications have been made.

16 **Table 2 & disc. Function – All of the variables listed in Table 2 are in the discriminant analysis which then can determine species ID at 100%, however, this then requires 3 different known feather types from an unknown individual bird in order to determine species? This is extremely unlikely in the pratical case of a feather found on the ground, in a criminal investigation or in an airplane strike. Does this disc. analysis work per feather type or what is the minimum number of features needed to accurately identify/categorize these two species?**

Indeed, obtaining multiple different known feather types (such as feathers found on the ground, in criminal investigations, or in an airplane strike) is extremely unlikely in practical cases. In these situations, a detailed analysis of a single feather type is necessary to accurately determine the species. Therefore, stepwise discriminant analysis was respectively carried out for all the microstructural characteristics of contour feathers, rectrices, and down feathers.

The barbule length, node number, and inter-node distance of down feathers, may be considered as crucial morphological markers for differentiating the Scaly-sided Merganser from the Common Merganser. For contour feathers, incorporating the base length into the model achieved a 56.9% correct classification rate. In rectrices, the hooklet number and prong length emerged as key discriminators, with a correct classification rate of 91.3%. Most notably, the barbule length, node number, and inter-node distance of down feathers demonstrated exceptional discriminative capabilities, attaining a perfect 100% correct classification rate. Consequently, the barbule length, node number, and inter-node distance of down feathers, may be considered as crucial morphological markers for differentiating the Scaly-sided Merganser from the Common Merganser.

17 **Line 262 – multiple features – which combination of features then are recommended to make this identification if the individual characters found in Table 1 (with significant differences between species) cannot be used.**

It has been modified and adjusted. We recommend the barbule length, node number, and inter-node distance of down feathers, may be considered as crucial morphological markers for differentiating the Scaly-sided Merganser from the Common Merganser.

18 **Line 271-272 – I’m not sure that this study on morphological barbule characters aids in the exploration of ecological characteristics, habitat, dietary preferences and breeding behaviors. I think that may be a bit of a stretch?**

We agree with your point, and we have removed the relevant content and made the necessary revisions.
