# Supplementary material for: Differences between Scaly-sided Merganser (Mergus squamatus) and Common Merganser (M. merganser) feather microstructure
Source: PLoS One. 2026 Jan 27;21(1):e0319144. doi: 10.1371/journal.pone.0319144 (PMC12843524; doi:10.1371/journal.pone.0319144)
Supplement: Attachment — Submitted filename: Response_to_Reviewers_auresp_2.docx [file pone.0319144.s004.docx]

**Response to Reviewers**

**1.The authors mention that flank contour feathers were included in the analysis; however, it is unclear how exactly the flank contour feathers were identified among the feathers provided by volunteers. This is particularly important for the Common Merganser (Mergus merganser), in which males have a large portion of the underside (including the breast and belly) covered with white plumage, similar in appearance to the flanks. This raises the risk of misidentifying the feather type during sampling, especially if the feathers were collected without access to the carcass. I recommend clarifying which morphological or anatomical criteria were used to identify flank feathers specifically, and how the possibility of including feathers from other similarly colored body areas (e.g., breast or belly) was excluded.**

We sincerely appreciate you for your insightful comments on the identification of flank contour feathers in our study. We acknowledge the challenge of distinguishing these feathers from other similarly coloured ones, especially in male common mergansers, which have white feathers on their breast, belly, and flanks. It should be emphasised that the feathers provided by a volunteer in Fuzhou were provided on the basis of anatomical position. On this basis, we checked these feathers against feathers in the herbarium. By observing the macrostructure of the feathers under the light microscope, we focused on morphological features such as the length, width, and curvature of the feather shaft. Flank contour feathers were found to be longer than those from the breast and belly, with a thicker and more curved shaft. These features differed significantly from feathers of other body parts, enabling us to effectively exclude feathers that might have been derived from other regions.

**2． It is unclear in what form the feathers were provided by the volunteers. If they were submitted as a general batch without separation by individual bird or by anatomical origin, there is a considerable risk that feathers not corresponding to the intended body region were included in the analysis, or that multiple feathers from the same individual were analyzed. This situation could distort within-group variability and lead to an overestimation of interspecific differences. A similar issue arises with the Scaly-sided Merganser samples, as the exact number of contributing individuals is also unknown. In this context, the reported classification accuracy—especially the 100% result for down feathers—raises concerns. While interspecific differences may indeed be present, I believe it is essential to perform additional validation of the model, for example, using cross-validation techniques, to rule out overfitting and confirm the robustness of the results.**

We apologize for the lack of clarity regarding the form in which feathers were provided by volunteers. Feathers donated by a volunteer in Fuzhou were collected based on anatomical position, and we have now explicitly stated this in the manuscript. Furthermore, all reported classification accuracies, including the 100% result for down feathers, were obtained using a leave-one-out cross-validation approach, a detail we have also added throughout the manuscript. These additions should address concerns about the robustness of our findings and potential overfitting of the model.

**3．The manuscript does not specify the anatomical region from which the down feathers were collected—despite the fact that these feathers yielded the highest classification accuracy (100%). Given that the microstructure of down feathers may vary depending on their position on the bird's body, the lack of this information complicates both the interpretation of the results and the reproducibility of the study. It is possible that the morphological features of downy barbules are consistent across body regions; however, before making generalized conclusions, it would be appropriate to investigate this question in more detail—or at the very least, to acknowledge it as a potential limitation of the method.**

**4. The authors state that they were unable to d etermine the age of the individuals from which the feathers were collected, and therefore performed the analysis without separating samples by age group. However, this approach leaves open the possibility of unequal age distribution between species. For example, the Common Merganser sample might include up to five juvenile individuals, while the Scaly-sided Merganser sample may include only one. Given that microstructural characteristics of feathers—including barbule morphology, node count, and hooklet number—can vary with age, such asymmetry could influence the results of interspecific comparisons. It is recommended that this factor be at least addressed as a potential limitation of the study, particularly in the context of interpreting the high accuracy reported in species-level classification.**

Thank you for your insightful feedback. We fully recognize the limitations of our study, primarily stemming from the lack of information regarding the collection sites of down feathers and the ages of sampled individuals. These gaps in data have the potential to influence feather microstructural characteristics, thereby affecting the generalizability and reliability of our results.

In response, we have added a detailed discussion of these limitations to the manuscript. Specifically, we note two key constraints. First, the specific collection areas of down feathers were not recorded. Given that down feathers from different body parts of birds may exhibit distinct microstructural characteristics due to functional differences, the lack of this information not only introduces uncertainties in the results but may also undermine the study's reproducibility and comparability across different research works. Although the down feather samples of Scaly-sided Mergansers in this study were randomly collected, theoretically covering various body parts, and the analysis of down feathers from different parts of two Common Merganser individuals revealed minimal microstructural variations within the species but significant differences compared to Scaly-sided Mergansers, the absence of collection location information may still interfere with the interpretation of the results. Second, the age of the sample individuals is unknown. Since feather microstructure may change with growth stages, age distribution biases between species could introduce systematic errors and compromise the reliability of interspecies comparisons.

To address these limitations, we propose that future research adopt a more comprehensive sampling strategy. This approach should include collecting feathers from defined anatomical regions (such as the chest, abdomen, and wings) and across diverse age groups (juveniles, sub-adults, and adults). By implementing these improvements, we aim to enhance the rigor of feather microstructure studies. We hope that our current findings, despite their limitations, can serve as a foundation for more robust investigations in this field.

**5．It would be helpful for international readers if the total number of feathers used in the study were stated more clearly, along with the number of each feather type analyzed per species. Currently, the relevant information is presented in a fragmented way across the text, which makes it difficult to reconstruct the structure of the dataset. Providing this information explicitly—either in a separate paragraph or in a table—would improve the clarity of the methodology and increase confidence in the results.**

We have addressed your feedback by introducing a dedicated section in the Methods section to explicitly report the total number of feathers (120 samples: 60 per species) and the distribution of feather types (20 samples each for flank contour feathers, rectrices, and down feathers per species). The sampling strategies for each species—including individual-specific collections and volunteer-donated samples—and sample preparation protocols are now clearly outlined in a structured paragraph, eliminating the previous fragmented presentation. This revision ensures that readers can readily reconstruct the dataset composition and methodology, enhancing the study’s clarity and replicability. By specifying the number of samples per feather type and species, as well as detailing the sampling and processing steps, we aim to provide a transparent foundation for our results and build confidence in the analytical framework. Thank you for your insightful suggestion, which has significantly improved the methodological description.
