# Supplementary material for: Differences between Scaly-sided Merganser (Mergus squamatus) and Common Merganser (M. merganser) feather microstructure
Source: PLoS One. 2026 Jan 27;21(1):e0319144. doi: 10.1371/journal.pone.0319144 (PMC12843524; doi:10.1371/journal.pone.0319144)
Supplement: Attachment — Submitted filename: Response_to_Reviewers_auresp_3.docx [file pone.0319144.s005.docx]

**Response to Reviewers**

1. **The lack of detail about the exact sample sizes also ties in with the lack of detail about collection localities and personnel, about which we have raised a query with you via emails from the editorial office.**

The details have been supplemented in the manuscript.

1. **The limitations of this study need to be further expanded on to make it clear what the practical application (forensic application as suggested by the authors intro) of using these measurements to identify unknown feathers is and sample size needs to be made more clear for each species and each feather type as also suggested in a previous reviewer comment.**

The limitations have been further expanded, and the sample size for each species and every feather type is now explicitly stated.

2. **As it stands, it is phrased in such a way that one could mistake these characters and measurements are clear and robust enough to differentiate the two species, reliably. The sample size is not robust enough, nor is the intraspecific variation in barbule characters described well enough to utilize these microscopic characters alone characters to identify these two species in a forensic context.**

Thank you for your thorough review and constructive comments. We fully agree that the original wording could easily lead readers to believe that the current sample size and barbule characters already suffice for species identification. To avoid any misinterpretation, we have systematically revised the relevant sections and softened the language accordingly. The specific changes are as follows:

Lines 39-41: “Consequently, the barbule length, node number, and distance between nodes of down feathers, may serve as potentially useful morphological markers for differentiating the Scaly-sided Merganser from the Common Merganser.”

Lines 267-272: “In light of the above limitations, relying solely on microstructural features such as the barbule length, node number, and distance between nodes of down feathers is insufficient to support accurate species identification between the two species. Nevertheless, these metrics can still be regarded as potential morphological cues for distinguishing between the Scaly-sided Merganser and the Common Merganser,”

Lines 296-298: “Consequently, the barbule length, node number, and distance between nodes of down feathers, may serve as potentially useful morphological markers for distinguishing between the two species.”

3. **The clarification/isolation and identification of their different feather types (contour, rectrices and down feathers) would be extremely useful in helping parse apart any differences between species and within species variation. The practical applications are numerous and very clearly and well stated. I think if the limitations are more clearly stated, this is a useful addition to the body of literature out there on microscopic identification of feathers. While stated mostly in the discussion a little expansion on these limitations may be helpful. We need to know individual variation in these characters (barbule length, how it relates to the number of nodes, does the distance between nodes vary along the length of a barbule, does the distance between nodes vary along where on the feather it is measured (basal barbules or more distal barbules)). Especially because of how overall less common this field of study is, it feels critical to outline these potential limitations for any new forensic scientist or solicitor/lawyer who may want to use these measurements to help identify an unknown feather – given the small sample size of individuals.**

The limitations have now been supplemented as follows: “Third, due to limitations in the experimental materials (unclear number of individuals), intraspecific variation in the barbule characteristics of the Scaly-sided Merganser and Common Merganser has not been explored. In light of the above limitations, relying solely on microstructural features such as the barbule length, node number, and distance between nodes of down feathers is insufficient to support accurate species identification between the two species. Nevertheless, these metrics can still be regarded as potential morphological cues for distinguishing between the Scaly-sided Merganser and the Common Merganser, and their diagnostic value needs to be confirmed by expanding the sample size, standardizing sampling procedures, and conducting statistical validation.”

4. **Additionally – a limitation on the forensic application of these data is important. When talking about stepwise discriminant analysis it is helpful to know what measurements are NEEDED to make a classification, and why a basic difference with statistically significant measurement of prong length isn’t sufficient to determine between the two species which do not have overlap in measurements (per table 2) and why the stepwise discriminant analysis is necessary – UNLESS we know that there is a lot of individual variation in those measurements and there actually is quite a bit of overlap along different areas of the feathers or different barbules from different areas of the bird.**

We agree that clarifying the rationale for adopting stepwise discriminant analysis in this study is important. The relevant section has been revised as follows (Lines 232–246): "The microstructure of feathers provides an important reference for the identification of bird species [1]. Although species identification may be achieved with a single feather characteristic in many situations, it is strongly advisable to utilize multiple characteristics [6]. In situations such as ground investigations, criminal cases, or airplane collision incidents, conducting a thorough analysis of multiple microstructural features from the same feather is essential to determine its species of origin. Even if a single parameter (barbule length) shows a statistically significant difference and no apparent overlap between species (Table 6), identification based solely on this parameter may still be insufficient due to potential intraspecific variation and spatial differences in feather microstructure across different body regions. Stepwise discriminant analysis can address this limitation by selecting and combining the most discriminatory variables, thereby reducing the risk of misclassification. Based on the above considerations, in this study, stepwise discriminant analysis was also conducted on all microstructural characteristics of contour feathers, rectrices, and down feathers, with the aim of improving the accuracy and reliability of species identification.”

1. **Abstract: “All the microstructural features of down feathers, particularly barbule length, may be considered as crucial morphological markers for differentiating the two species.” Whereas the morphological barbule characters described as capable of discerning Scaly-sided and Common Merganser are barbule length, node number, and inter-node distance of down feathers, …for differentiating the Scaly-sided Merganser from the Common Merganser. Update to include only the relevant morphological characters/take home characters suggested/recommended to distinguish these two species.**

The abstract already states: “Consequently, the barbule length, node number, and distance between nodes of down feathers, may serve as potentially useful morphological markers for differentiating the Scaly-sided Merganser from the Common Merganser.” This sentence appears on Lines 39–41.

2. **It is mentioned that approx. 20 individuals for Scaly-sided Mergansers were sampled and the authors have given context that there were at least 20 individuals in the flock observed and later collected feathers from. It’s important to understand that this does not mean that all 20 individuals were sampled and should not be phrased as such without genetic confirmation of the minimum number of individuals that these feathers could have originated from. The paper stating that 20 individuals of this species are represented here is stating the maximum number of individuals the feathers could originate from, which is very likely an overestimate. For Common Merganser, it now appears that you have at least 3 individuals represented, but there is no way to claim that there were 20 Common Mergansers represented by the 2 known carcasses (ad. Female, ad male) and the multiple feathers from volunteer. The claim that this would represent at least 20 individuals is misleading and an overrepresentation. Please make explicit the sample size for each species (****Common Merganser,** **min number of individuals represented = 3) or (Scaly-sided Merganser, min number of individuals represented = X?) and then you can also detail that pooled by species at least 20 feathers of each feather type for each species were examined for this study. This would help clarify how many feathers were examined and from the minimum number of birds those feathers originated. Very helpful information to understand the breadth of representation in possible feather variation captured by this sampling.**

Already supplemented and revised: “A total of 120 feather samples were included in this study, representing a minimum of 5 Scaly-sided Mergansers and 3 Common Mergansers.”

**3. Line 232 – Common Merganser, the C in Common should be capitalized.**

Already corrected.
