# Supplementary material for: Differences between Scaly-sided Merganser (Mergus squamatus) and Common Merganser (M. merganser) feather microstructure
Source: PLoS One. 2026 Jan 27;21(1):e0319144. doi: 10.1371/journal.pone.0319144 (PMC12843524; doi:10.1371/journal.pone.0319144)
Supplement: Attachment — Submitted filename: Response_to_Reviewers_auresp_4.docx [file pone.0319144.s006.docx]

**Response to Reviewers**

**Reviewer #4:**

1. **Lines 89-90: The sentence “A group of more than 20 Scaly-sided Mergansers was observed through binoculars during the moulting period.” is not precise enough. As far as I know, for the Scaly-sided Mergansers, at least adult males do not molt during the overwintering period, but during the breeding period when females hatch.**

We sincerely appreciate the reviewer for pointing out this critical oversight, which improves the scientific rigor of our manuscript. We have revised the inaccurate description in the Materials and Methods section (Lines 89–90), correcting “during the moulting period” to “during the field survey”.

2. **Line 130: Please replace “320X to 2600X” with “320× to 2600×”.**

Already corrected.

3. **Lines 167-168: The table 3 is redundant, should be deleted. Please include the information of table 3 in the contents of results section.**

Thank you very much for your valuable suggestions. Tables 3, 5, and 7 in the paper constitute a set of corresponding analytical results, which respectively focus on the stepwise discriminant analyses of the two species for contour feathers, rectrices, and down feathers. Removing only Table 3 might disrupt the coherence of the overall structure of the paper. Therefore, we intend to retain Table 3, and we would appreciate it if you could reconsider this arrangement.

4. **Discussion section: I recommend adding a brief discussion on why contour feathers, rectrices, and down feathers vary in their effectiveness for species identification.**

We have incorporated the recommended discussion in the revised manuscript (Lines 254–259), as follows: This discrepancy in taxonomic resolution across feather types presumably reflects contrasting functional constraints. Contour feathers and rectrices are strongly molded by ecological demands such as flight and display, predisposing them to convergent evolution [24]. Down feathers, specialized primarily for thermoregulation, experience relaxed selective pressure, retain phylogenetically conserved microstructures, and preserve greater species-specific information [25].

**Reviewer #5:**

1. **Please clarify the contour feathers are flight feathers or coverts?**

Thank you for your question. As noted in the Sample Preparation section (Lines 111), the contour feathers in this study specifically refer to flank contour feathers, which are neither flight feathers nor coverts in the strict sense.

2. **Is there an intraspecific difference in the ultrastructure of the three types of feathers of the same species?**

Thank you for this insightful question. As stated in lines 171–173, owing to the limited and poorly documented sample size (number of individuals unknown), we were unable to examine intraspecific variation in the ultrastructure of the three feather types within either the Scaly-sided Merganser or the Common Merganser. This aspect will be addressed once additional specimens of known provenance become available.

3. **Reference 5: The format of capitalization for scientific names in the titles of references must be consistent with the format of other references.**

Already corrected.

4. **Reference 18: “cairina to mergus”中的“cairina” “mergus” should be italicized.**

Already corrected.
